# Compliance with standard precaution practices and associated factors among health care workers in Dawuro Zone, South West Ethiopia, cross sectional study

**DOI:** 10.1186/s12913-019-4172-4

**Published:** 2019-06-13

**Authors:** Abera Beyamo, Terefe Dodicho, Wolde Facha

**Affiliations:** Public Health Department, College of Health Sciences, Wolaita Sodo University, Wolaita Sodo, Ethiopia

**Keywords:** Standard precaution practice, Health care workers

## Abstract

**Background:**

Infection in healthcare facilities is a major public health problem in most developing countries like Ethiopia. Currently the overall incidence of health care associated infection has been increased and burden of these infections is staggering. This study aimed to assess health care workers compliance with standard precaution practices and associated factors in public health institutions of Dawuro zone, south west Ethiopia, 2016.

**Methods:**

An institution based cross sectional study was conducted from February1–30/ 2016 in 17 health institutions found in Dawuro zone. Data were collected using a pre-tested questionnaire on 250 health care workers selected by simple random sampling technique. Collected data was checked, coded and analyzed by using SPSS version 20. A *P*-value less than 0.05 was considered as statistically significant.

**Result:**

Two hundred fifty health care workers were participated in the study. Out of the total respondents, 162(65.0%) of respondents had complied with standard precaution practices. Service year less than or equal to 5 years, training on standard precaution, having good hand hygiene and availability of personal protective equipment were independently associated with compliance to standard precaution practices.

**Conclusion:**

Significant numbers of health care workers were not complied with standard precaution practices in this study. Therefore strategies targeted in fulfilling health institutions with supplies like hand hygiene material, Personal Protective equipment and training on standard precaution will improve compliance with standard precaution practice.

**Electronic supplementary material:**

The online version of this article (10.1186/s12913-019-4172-4) contains supplementary material, which is available to authorized users.

## Background

Standard Precautions are work practices required to achieve a maximal level of infection control for the treatment of all clients regardless of diagnosis. It refers to all policies, procedures and activities which aim to prevent or minimize the risk of transmission of infectious disease at health care institutions. The use of standard precautions is recommended for all patients, regardless of suspected or confirmed infection status [1–3]. Standard precautions includes hand hygiene (routine hand washing, hand antisepsis, and surgical hand scrub), personal protective equipment (capes, gowns, masks, aprons, drapes, closed boots or shoes, goggles or glasses, sterile drapes), prevention of needle stick or sharp injury, waste management, instrument processing (decontamination, cleaning,sterilization), processing linen, housekeeping and clinical laboratory services [1, 4–9]. Occupational exposure to blood and body fluids is a serious concern for health care workers and presents a major risk for the transmission of infections such as human immune-deficiency virus (HIV), hepatitis B virus (HBV), and hepatitis C virus (HCV) [2, 10, 11]. Developing countries, especially those in sub-Saharan Africa, that account for the highest prevalence of HIV-infected patients in the world also report the highest incidences of occupational exposures. Unless appropriate standard precautions are in practice, health care facilities can be the source of infection and epidemic disease for the community at large. Very few studies were conducted in Ethiopia to assess compliance to standard precaution practices among health care workers and factors inhibiting the practice. So this study aimed to assess health care workers compliance with standard precaution practices and associated factors in public health institutions of Dawuro zone.

## Methods

### Study design period and area

An institution based cross-sectional survey was carried out from March 1–30, 2016 in 17 health institutions of Dawuro zone. The study was carried out in Dawuro zone, SNNPR, located in the South western part of Ethiopia, 515 km away from Addis Ababa. There were 1089 health care workers who had been working in the health institutions of the zone during the study period. The study included two hospitals (Tarcha General Hospital and Gessa district Hospital) and 15 health centers.

### Study population and sampling

All health care workers who were giving health care services in Dawuro Zone were considered as source population and sampled health care workers who were giving the service in the zone were taken as study population. Sample size was calculated by using single population proportion formula based on the following parameters: 95% confidence level (1.96), margin of error (0.05) and 22% (17) proportion of compliance with standard precautions. The calculated sample size was 256 health care providers working in the health institutions of the zone. A Sampling frame of each health care worker was prepared and proportional allocation to the size of study participants to each Hospitals and Health centers was employed.

### Data collection instrument and data collectors

A quantitative method of data collection was employed for assessment of compliance to standard precaution practice of health care workers. A face to face interviewer administered questionnaire and observational checklist that is adapted from different literatures and infection prevention guidelines were used for data collection [12]. The tool has three sections such as socio-demographic characteristics of the respondents (7 questions), individual related characteristics (5 questions) and health institution related characteristics (3 questions) (Additional file 1). Compliance to standard precautions practices was determined using questions related to Practices of health care workers on standard precaution. Rating questioners were included from 1 to 5 (1-never, 2-Seldom, 3-Sometimes, 4-Often, 5-Very often). Study participants scoring more than or equal to mean score were considered as having good compliance (complaints) to standard precaution practices and scores less than mean score were considered as poor compliance (none-compliant) to standard precaution practices. Four diploma nurses were participated in data collection and two BSc nurses were participated supervision process respectively.

### Data processing

The quality of data was assured by proper designing and pre-testing of the questionnaires to ensure consistency. The pre-test was conducted on 5% of the calculated sample at Wolaita Sodo University teaching and referral Hospital and Sodo Health center by data collectors and supervisors. Two days training were given for both data collectors and supervisors by the principal investigators. The training included discussion on the objectives of the study, contents of the questionnaires, data collection techniques and issues of confidentiality of the responses. Questionnaires were reviewed and checked for completeness by the supervisors and the principal investigators and the necessary feedback was offered to data collectors throughout the study daily. All the questionnaires were checked visually, coded and entered into EpiData version 3.1 software and were exported to SPSS version 20 software packages for analysis. The pre-test data was not included in the main data analysis (Additional file 1). 

### Data analysis

Descriptive analysis was carried out for each of the variables to check frequency, distribution and missing value. Bivariate logistic regression analysis was employed to check crude association between compliance to standard precaution practice and independent variables. Variables with *p* value < 0.25 on bivariate logistic regression analysis was entered in to multivariable logistic regression to identify the factors that affect Compliance to standard precaution practice. Odds ratio and corresponding 95% confidence intervals was used to quantify the degrees of association between independent variable and the outcome variable. Results with *p*-value < 0.05 were considered as being statistically significant and the rest was refuted. Multicollinearity among independently associated variables was checked by multicollinearity diagnostic test VIF in linear regression and none was collinear.

## Results

### Socio-demographic characteristics

From the study participants 131(52.4%) were within the age range 25–30, 163(65.2%) were females, 158(63.2%) were none-technical workers, 175(70.0%) were with degree and above educational status, 145(58.0%) were having 2–5 year service, 105(42.0%) were from Tercha Hospital and 183(73.2%) were married. (See Table 1).

**Table 1:** Socio-demographic characteristic of the study participants in Dawuro zone, south west Ethiopia, 2016

Explanatory Variables	Number	Percent
Age		
< 25 ears	28	11.2
25–30	131	52.4
≥ 31	91	36.4
Sex		
Male	87	34.8
Female	163	65.2
Profession		
Technical	92	36.8
Non – technical	158	63.2
Educational status		
Diploma and below	75	30.0
Degree and above	175	70.0
Years of service delivery		
< 2 years	32	12.8
2–5 years	145	58.0
≥ 6 years	123	49.2
Level of institution		
Tercha hospital	105	42.0
Gessa hospital	47	18.8
Health centres	98	39.2
Marital status		
Married	183	73.2
Divorced	6	2.4
Widowed	2	0.8
Single	59	23.6

### Proportion of precaution

A total of 250 health care workers participated in the study and 162(54.0%) health care workers complied with standard precaution practices.

### Individual characteristics of health care workers

Out of 250 study participants 145(58.0%), of them have good hand hygiene practice, 218(87.2%) have good PPE practice, 222(88.8%) have good injection safety practice, 169(67.6%) have good handling sharps practice and 202(80.8%) have good instrument processing and waste management. (See Table 2).

**Table 2:** Individual characteristics of health care workers on compliance with standard Precaution practice in Dawuro zone, South west Ethiopia, 2016

Explanatory Variables	Number	Percent
Hand hygiene practice		
Good	145	58.0
Poor	105	42.0
PPE practice		
Good	218	87.2
Poor	32	12.8
Injection safety practice		
Good	222	88.8
Poor	28	11.2
Handling sharps practice		
Good	169	67. 6
Poor	81	32.4
Instrument processing and waste management		
Good	202	80.8
Poor	48	19.2

### Health institution related factors

From a total of 250 study participants 150(60.0) were within the institutions having personal protective equipment (PPE) 130(52.0%) were having training on standard precautions and 135(54.0%) were having monitoring and evaluation on standard precautions. (See Table 3).

**Table 3:** Health institution related factors of health care workers on compliance with standard precaution practice in Dawuro zone, South west Ethiopia, 2016

Explanatory Variables	Number	Percent
Availability of PPE		
Yes	150	60.0
No	100	40.0
Training on standard Precautions		
Yes	120	48.0
No	130	52.0
Monitoring and Evaluation on standard precautions		
Yes	135	54.0
No	115	46.0

### Bivariate binary logistic regression analysis of associated factors with compliance with standard precaution among health professionals of Dawuro Zone, South West Ethiopia

The bivariate analysis revealed that age, service year, hand hygiene practice, availability of PPE to apply standard precaution and training on standard precaution were candidates for further analysis in multivariable logistic regression. (See Table 4).

**Table 4:** Bivariate binary logistic regression analysis of factors associated with compliance to standard precaution practice in Dawuro zone, South west Ethiopia, 2016

Explanatory variables	Compliance to standard precaution practice			
	Good compliance	Poor compliance	COR (95%Cl)	*p*-value
Age category				
< 25 years	12 (4.8)	16 (6.4)	0.27 (0.12–0.63)	0.003
25–30 Years	91 (36.4)	40 (16.0)	0.57 (0.32–0.92)	0.020
≥ 31 years	59 (23.6)	32 (12.8)	1	
Sex				
Male	65 (26.0)	22 (8.8)	1	
Female	97 (38.8)	66 (26.4)	0.26 (0.15–0.45)	< 0.001
Profession				
Technical	85 (34.0)	7 (2.8)	11.46 (5.06–25.96)	< 0.001
Non-Technical	77 (30.8)	81 (32.4)	1	
Education Status				
Diploma and below	1 (0.4)	74 (29.6)	1	
Degree and above	161 (64.4)	14 (13.6)	43.5 (58.47–57.47)	0.051
Years of service delivery				
≤ 5 years	61 (24.4)	66 (24.6)	1.85 (0.74–4.63)	0.19
> 5 years	81 (32.4)	42 (16.8)	1	
Level of health institutions				
Tarcha General hospital	62 (24.8)	43 (17.2)	0.68 (0.41–1.13)	0.34
Gessa district hospital	36 (14.4)	11 (4.4)	0.68 (0.30–1.58)	0.38
Health center	14 (45.6)	84 (38.4)	1	
Hand hygiene practice				
Good	137 (54.8)	8 (3.2)	12.13 (6.88–21.37)	0.0001
Poor	25 (10.0)	80 (32.0)	1	
Handling sharps practice				
Good Practice	117 (46.8)	52 (20.8)	0.80 (0.50–1.28)	0.35
Poor Practice	25 (10.0)	56 (22.4)	1	
Instrument processing & waste mgt				
Good practice	184 (73.6)	18 (7.2)	0.01 (0.001–2.02)	0.41
Poor practice	8 (3.2)	40 (16.0)	1	
Availability of PPE				
Yes	100 (40.0)	50 (20.0)	0.29 (0.17;0.48)	0.0001
No	62 (24.8)	38 (15.2)	1	
Training on standard precautions				
Yes	93 (37.2)	27 (10.8)	3.47 (2.06;5.84)	0.0001
No	69 (27.6)	61 (24.4)	1	
Monitoring and Evaluation on standard precautions				
Yes	122 (48.8)	13 (5.2)	1.92 (0.98;3.78)	0.56
No	40 (16.0)	75 (30.0)	1	

### Factors independently associated with compliance with standard precaution

In this study age below 25 years were 73% less likely comply than those with age > =31 years (AOR = 0.27, 95%CI (0.12,0.63) and age 25–30 years were nearly three times more likely comply than those with age > = 31 years (AOR = 2.57, 95%CI (1.32,3.92),service year ≤5 year were nearly nine times more likely comply with standard precaution than these with service year greater than 5 years (AOR = 8.55(6.72,12.25), availability of PPE to apply standard precautions were nearly ten times more likely to comply with standard precaution than the absence of PPE (AOR = 10.29, 95%CI (8.17,14.48), and health care workers getting training on standard precaution were twelve times more likely comply with standard precaution than those did not get training (AOR = 12.13, 95%CI (8.88,13.37). (see Table 5).

**Table 5:** Multivariable analysis of factors associated with Compliance with standard precaution practice in Dawuro zone, South west Ethiopia, 2016

Explanatory variables	Compliance to standard precaution practice				
	Good compliance	Poor compliance	COR (95%Cl)	AOR (95%CI)	*p*-value
Age category					
< 25 years	12 (4.8)	16 (6.4)	0.27 (0.12–0.63)	0.27 (0.12,0.63)	0.003*
25–30 Years	91 (36.4)	40 (16.0)	0.57 (0.32–0.92)	2.57 (1.32,3.92)	0.002*
≥ 31 years	59 (23.6)	32 (12.8)	1		
Service year					
≤ 5 years	61 (24.4)	66 (24.6)	1.85 (0.74–4.63)	8.55 (6.72,12.25)	0.001*
> 5 years	81 (32.4)	42 (16.8)	1	1	
Hand hygiene practice					
Good practice	137 (54.8)	8 (3.2)	0.29 (0.17;0.48)	2.45 (0.87,5.50)	0.061
Poor practice	25 (10.0)	80 (32.0)	1	1	
Availability of PPE					
Yes	100 (40.0)	50 (20.0)	0.29 (0.17;0.48)	10.29 (8.17,14.48)	0.001*
No	62 (24.8)	38 (15.2)	1	1	
Training on standard precautions					
Yes	93 (37.2)	27 (10.8)	3.47 (2.06;5.84)	12.13 (8.88,13.37)	0.001*
No	69 (27.6)	61 (24.4)	1	1	

## Discussion

In this study, 35% of health care workers were not compliant with standard precaution practices whereas 65% of health care workers complied with standard precaution practices. This finding is different from the study conducted in Mekele which showed that 43% of health care workers complied with standard precaution practices [13]. This might be due to differences in socio demographic factors and accessibility to adequate supply of equipment. This study showed that health care workers with less than or equal to 5 years service were 2.5 times more likely complied with standard precaution practice compared to those health care workers with greater than five service years. This finding is in line with study done in Mekele [13, 14]. This might be due to recent memory, strong commitment and fear of nosocomial infection. This finding is inconsistent with the study done in Bihar Dar; in which health care workers who had working experience greater than10years complied with standard precaution practices 1.48 times higher than their counterparts [4, 14]. This discrepancy might be due to the greater compliance to standard precaution practices among health care workers with longer years of experience due to their participation in a greater number of seminars, conferences and training which include standard precaution practices which not only encouraged safer work practices but also improved concordance with policy and procedures. In this study, 137(54.8%) of health care workers who complied with standard precaution practices had practiced hand hygiene techniques. This finding is lower than study done in Pune (India) in which 85% of health care workers who complied with standard precaution practices had practiced hand hygiene techniques [15].

### Recommendation

#### Zonal health department, hospital and health centers

Improving the availability of supplies like hygienic hand washing material with personal protective equipments, water supply with different sterilizers and disinfectants in order to improve compliance with standard precaution practice.

Giving Pre-service training with adequate time and durations for immediate engagement of the new employee to standard precaution practice will improve standard precaution practice.

#### Researchers

To conduct prospective study regarding factors affecting health care workers compliance with standard precaution.

## Conclusion

In this study, a total of 250 health care workers participated in the study and 88(35%) and 162(65%) health care workers poorly complied and well complied with standard precaution practices respectively. From this study, we concluded that those health care workers who had service year ≤5 years, the age group 25–30 years, health care workers who reported having PPE in their health institutions and those who got training on standard precaution were compliant with standard precaution practices. Majority of the study participants who were compliant with standard precaution practices were married, females, technical staffs, and those who had degree and above educational status.

## Additional file

Additional file 1: English version Interviewer administered Questionnaire _ Observation checklist. Compliance with Standard Precaution Practices and Associated Factors among Health Care Workers. Interviewer administered Questionnaire and Observation checklist to assess the compliance with Standard Precaution Practices and Associated Factors among Health Care Workers. (DOCX 14 kb)

## Data Availability

The data that support the findings of this study are available but some restrictions may apply to the availability of these data as there are some sensitive issues. However, data are available from the corresponding authors upon reasonable request.

## Abbreviations

BSc: Bachelor of Science
HBV: Hepatitis B Virus
HCV: Hepatitis C Virus
PPE: Personal Protective Equipment
SPSS: Statistical Package for Social Science
