# Supplementary material for: Compliance with standard precaution practices and associated factors among health care workers in Dawuro Zone, South West Ethiopia, cross sectional study
Source: BMC Health Serv Res. 2019 Jun 13;19:381. doi: 10.1186/s12913-019-4172-4 (PMC6567427; doi:10.1186/s12913-019-4172-4)
Supplement: Supplementary file 1 — English version Interviewer administered Questionnaire _ Observation checklist. Compliance with Standard Precaution Practices and Associated Factors among Health Care Workers. Interviewer administered Questionnaire and Observation checklist to assess the compliance with Standard Precaution Practices and Associated Factors among Health Care Workers. (DOCX 14 kb) [file 12913_2019_4172_MOESM1_ESM.docx]

# Questionaire: English version

Informed Consent Form for Quantitative face to face interview questionnaires:

100A. Name of health institution

100B. Date_____________ 100C. Time started--------------------

100D. Department------------------------------------ 100E. Code number of the checklist

Hallo! Good morning?

My name is Sr./ Ato ------------------------------------------.

Today I am here to collect data on "the assessment of compliance to standard precaution practices and risk factors associated with it".

I would like to assure you that the study is confidential. I will not keep a record of your name and address. You have a right to stop the interview at any time, or to skip any question that you do not want to answer. Your correct answer to the questions can make the study achieve the goals. Therefore, you are kindly requested to respond genuinely and voluntary with patience. The interview may take about ----------- minutes.

Do you have any question?

Are you willing to participate in the interview?

[ ] Yes, Go to the next page

[ ] No, Thank them and interrupt the interview

Signature of the consenting interviewer-------------------------------------------

100F.Result of the interview: 1. Completed 2.Partially completed 3. The interviewee refused

4. Others--------------

Data collector’s Name:----------------------------- Signature ---------------

Supervisor’s name-------------------------------- Signature -----------------

Part-1.Socio-demographic factors (Give response by "*circling*" the number)

| S/No | Question Related to knowledge. | Response |
| --- | --- | --- |
| 101 | Level of Health Institution | 1. Zona l hospital 2. District Hospital 3. Health center |
| 102 | Age s | ------------------- |
| 103 | Sex | 1. 1. Male 2. Female |
| 104 | Profession | 1.Surgeon 2.Internist 3.Obs&gynacologist 4.Pediatricians 5.Dentistry 6.GP  7.Health Officer 8.Bsc Nurse 9.Diploma Nurse 10.Midwives of all types 11.Lab technicians 12.Housekeeping personnel 13.Laundry personnel |
| 105 | Education Status | 1.Grade1-8 1. 2 .Grade 9-12 3.Diploma and above |
| 106 | Years of service | ----------------------------------------------- |
| 107 | Marital status | 1. Single 2. Married 3. Separated 4. Widowed |

Part-2. Health institution factors. (Give response by "*circling*" the number)

| S/  No | Question | Response | | | | Skip To |
| --- | --- | --- | --- | --- | --- | --- |
|  |  | Yes | No | Not Available | I do not know |  |
| 208 | Is there enough supplies to apply standard precautions? | 1 | 2 | 88 | 99 | 2🡪212  88🡪212  99🡪212 |
| 209 | If "1" ,what are they? |  |  |  |  |  |
|  | 1.water | 1 | 2 | 88 | 99 |  |
|  | 2.Soap | 1 | 2 | 88 | 99 |  |
|  | 3.Alcohol | 1 | 2 | 88 | 99 |  |
|  | 4.others | 1 | 2 | 88 | 99 |  |
| 210 | Did you take training on standard precautions? | 1 | 2 | 88 | 99 | 2or88or99🡪214 |
| 211 | If" 1 "on which standard precautions did you take training? |  |  |  |  |  |
|  | 1.Hand hygiene | 1 | 2 | 88 | 99 |  |
|  | 2.Personal protective equipment | 1 | 2 | 88 | 99 |  |
|  | 3.Safe injection practices | 1 | 2 | 88 | 99 |  |
|  | 4.Handling and disposing sharps | 1 | 2 | 88 | 99 |  |
|  | 5.instrument processing and waste managements | 1 | 2 | 88 | 99 |  |
| 212 | Is there M&E on standard precautions? | 1 | 2 | 88 | 99 |  |

Part-3.Individual characteristics (Give response by "*circling*" the number)

| S/  No | Question | Response | | | Ski skip |
| --- | --- | --- | --- | --- | --- |
|  |  | Yes | No | I do not know |  |
| 313 | Do you know when you have to wash your hands? | 1 | 2 | 99 | 2 or 99-->317 |
| 314 | If ''1'' When do you wash your hands? |  |  |  |  |
|  | 1. Before commencing the Procedure | 1 | 2 | 99 |  |
|  | 2.Before any contacts | 1 | 2 | 99 |  |
|  | 3 Before completing the procedure | 1 | 2 | 99 |  |
|  | 4.After any contacts | 1 | 2 | 99 |  |
| 315 | Do you know what can be used to keep your hand hygiene? | 1 | 2 | 99 | 2 or99🡪319 |
| 316 | If"1"What do you use to keep your hand hygiene? |  |  |  |  |
|  | 1. Plain water | 1 | 2 | 99 |  |
|  | 2. Antimicrobial soap and water | 1 | 2 | 99 |  |
|  | 3.Any Alcoholic solutions | 1 | 2 | 99 |  |
|  | 4. Surgical hand scrub | 1 | 2 | 99 |  |
|  | 5.Any water | 1 | 2 | 99 |  |
| 317 | Can you list Hand hygiene techniques? | 1 | 2 | 99 | 2 or99🡪321 |
| 318 | If"1"what are they? |  |  |  |  |
|  | 1. Routine Hand washing | 1 | 2 | 99 |  |
|  | 2.Using gloves and hand antisepsis | 1 | 2 | 99 |  |
|  | 3. Antiseptic Hand rub | 1 | 2 | 99 |  |
|  | 4. Surgical Hand scrub and using surgical gloves | 1 | 2 | 99 |  |
| 3 19 | Can you mention the Steps that increase the chances of success of the  staff towards hand washing? | 1 | 2 | 99 | 2 or99🡪323 |
| 320 | If"1"wha are they? |  |  |  |  |
|  | 1. Widely disseminating current guidelines for hand hygiene practices | 1 | 2 | 99 |  |
|  | 2. Making available alternative options like waterless alcohol-based hand rubs. | 1 | 2 | 99 |  |
|  | 3.Strong punishments. | 1 | 2 | 99 |  |
| 321  322 | Do you know the aim of standard precaution? | 1 | 2 | 99 | 2 or99🡪325 |
|  | If"1"what are they? |  |  |  |  |
|  | 1. protecting Health care workers from getting infections from patients | 1 | 2 | 99 |  |
|  | 2.to prevent patients getting infected from Health care workers | 1 | 2 | 99 |  |
|  | 3.aimed only to protect Health care workers from infections | 1 | 2 | 99 |  |
|  | 4.to get incentives | 1 | 2 | 99 |  |
| 323 | Do you know Personal protective equipments? | 1 | 2 | 99 | 2 or99🡪327 |
| 324 | If"1"what are they? |  |  |  |  |
|  | 1.Gloves | 1 | 2 | 99 |  |
|  | 2.spread sheets | 1 | 2 | 99 |  |
|  | 3.Gowan | 1 | 2 | 99 |  |
|  | 4.shirts | 1 | 2 | 99 |  |
| 325 | Can you tell when do you use Personal protective equipments? | 1 | 2 | 99 | 2 or99🡪329 |
| 326 | If"1"when do you use them? |  |  |  |  |
|  | 1.before touching anything potentially infectious and wet | 1 | 2 | 99 |  |
|  | 2.After touching broken skin, mucous membrane, blood, body fluids,  secretions or excretion or soiled instrument | 1 | 2 | 99 |  |
|  | 3.before performing invasive procedures. | 1 | 2 | 99 |  |
|  | 4.Before touching any person | 1 | 2 | 99 |  |
| 327 | Do you know the types of gloves that can be used during  surgical procedure? | 1 | 2 | 99 | 2 or99🡪331 |
| 328 | If"1"what are they? |  |  |  |  |
|  | 1.Disposable clean examination gloves | 1 | 2 | 99 |  |
|  | 2.Sterile surgical glove | 1 | 2 | 99 |  |
|  | 3.High-level disinfected surgical gloves | 1 | 2 | 99 |  |
|  | 4.Utility gloves | 1 | 2 | 99 |  |
|  | 5.Any available gloves | 1 | 2 | 99 |  |
| 329 | Do know how do you prevent needle stick or sharp injuries? | 1 | 2 | 99 | 2 or99🡪333 |
| 330 | If"1"how do you prevent needle stick or sharp injuries? |  |  |  |  |
|  | 1. by open damping | 1 | 2 | 99 |  |
|  | 2. disposing or sharps in puncture resistant containers | 1 | 2 | 99 |  |
|  | 3.Avoiding Work loaded | 1 | 2 | 99 |  |
|  | 4.Reusing needle and syringes. | 1 | 2 |  |  |
|  | 5.Avoid Recapping needle and syringes | 1 | 2 | 99 |  |
|  | 6 .By using safety box and other open container | 1 | 2 | 99 |  |
|  | 7. safely passing sharp instruments | 1 | 2 | 99 |  |
| 331 | Are you familiar with the method to Prevent Contamination of  Injection Equipment and Medication | 1 | 2 | 99 | 2 or99🡪335 |
| 332 | If"1"what are they? |  |  |  |  |
|  | 1. Use multi-dose vials rather than single dose vials | 1 | 2 | 99 |  |
|  | 2. Swabbing of a new vial tops with an antiseptic or disinfect is unnecessary. | 1 | 2 | 99 |  |
|  | 3. Skin preparation that is visibly soiled with soap and water before injection. | 1 | 2 | 99 |  |
|  | 4. Discard a needle that has touched any surface. | 1 | 2 | 99 |  |
| 333 | Can you list the types of sharp collection materials? | 1 | 2 | 99 | 2 or99🡪343 |
| 334 | If"1"which of the followings? |  |  |  |  |
|  | 1. Safety box/ card box/ | 1 | 2 | 99 |  |
|  | 2.Plastic pail with lid | 1 | 2 | 99 |  |
|  | 3.Plastic pail without lid | 1 | 2 | 99 |  |
|  | 4.Any open container. | 1 | 2 | 99 |  |
| 335 | Can you mention methods which Prevent Access  to Used Needles and Syringes? | 1 | 2 | 99 | 2 or99🡪339 |
| 336 | If"1"what are they? |  |  |  |  |
|  | 1. Seal sharp containers for transport during disposal | 1 | 2 | 99 |  |
|  | 2. put sharps containers close to the point of use within arm’s reach | 1 | 2 | 99 |  |
|  | 3. Shake a container to settle its contents and make room for more sharps. | 1 | 2 | 99 |  |
|  | 4. Do mark the fill line at the three quarters full level. | 1 | 2 | 99 |  |
| 337 | Do you know the advantage of waste management? | 1 | 2 | 99 | 2 or99🡪341 |
| 338 | If"1"what are they? |  |  |  |  |
|  | 1. to protect people from accidental injury, | 1 | 2 | 99 |  |
|  | 2. to prevent the spread of infection to healthcare workers. | 1 | 2 | 99 |  |
|  | 3. to prevent the spread of infection to the local community | 1 | 2 | 99 |  |
|  | 4. to safely dispose of hazardous materials | 1 | 2 | 99 |  |
| 339 | Can you list the types waste materials that generate from  health institutions? | 1 | 2 | 99 | 2 or99🡪343 |
| 340 | If"1" what are they? |  |  |  |  |
|  | 1. Non-contaminated wastes | 1 | 2 | 99 |  |
|  | 2.Contaminated waste | 1 | 2 | 99 |  |
|  | 3.Sterile wastes | 1 | 2 | 99 |  |
| 341 | Are you familiar with the steps of waste management? | 1 | 2 | 99 | 2 or99🡪345 |
| 342 | If"1"what are they? |  |  |  |  |
|  | 1. Segregation | 1 | 2 | 99 |  |
|  | 2.Opn damping | 1 | 2 | 99 |  |
|  | 3.Decontamination | 1 | 2 | 99 |  |
|  | 4.Disposal | 1 | 2 | 99 |  |
| 343 | The function of" Incineration" is non controlled burning of solid, liquid or gaseous combustible wastes . | 1 | 2 | 99 |  |
| 344 | Are familiar with the steps in processing contaminated instruments  ,and other Items? | 1 | 2 | 99 | 2 or99🡪348 |
| 345 | If"1"what are they? |  |  |  |  |
|  | 1.decontamination (soak in 0.5% chlorine solution for 10 minute) | 1 | 2 | 99 |  |
|  | 2. Disposal | 1 | 2 | 99 |  |
|  | 3. Sterilization | 1 | 2 | 99 |  |
|  | 4.High-level disinfection | 1 | 2 | 99 |  |
|  | 5.Segreggation | 1 | 2 | 99 |  |
| 346 | Do you know from where health institution wastes are generated? | 1 | 2 | 99 | 2 or99🡪350 |
| 347 | If"1"from where? |  |  |  |  |
|  | 1.Used materials from health care providers | 1 | 2 | 99 |  |
|  | 2.Pharmacitical waste | 1 | 2 | 99 |  |
|  | 3.Patients waste | 1 | 2 | 99 |  |

| S/No | Questions related to Attitudes | Responses | | | | |
| --- | --- | --- | --- | --- | --- | --- |
|  |  |  |  |  |  |  |
|  |  | Very dissatisfid=1 | Dissatisfied=2 | Neutral=3 | Satisfied=4 | very satisfied=5 |
| 348 | A non irritating, antiseptic hand rub can be made by adding either glycerin, propylene glycol or sorbitol to alcohol. | 1 | 2 | 3 | 4 | 5 |
| 349 | Preference to wash hands before and after the procedure. | 1 | 2 | 3 | 4 | 5 |
| 350 | Telephones and door knobs are not source of infections. | 1 | 2 | 3 | 4 | 5 |
| 351 | Removing rings, watches and bracelets is sometimes appropriate in surgical hand scrub. | 1 | 2 | 3 | 4 | 5 |
| 352 | Using personal protective equipment is not an easy task. | 1 | 2 | 3 | 4 | 5 |
| 353 | Using PPE harm patients psychologically, so do not use it | 1 | 2 | 3 | 4 | 5 |
| 354 | Don't use latex gloves if you have allergy to latex | 1 | 2 | 3 | 4 | 5 |
| 355 | Do keep fingernails trimmed moderately short to reduce the risk of tearing gloves. | 1 | 2 | 3 | 4 | 5 |
| 356 | Gloves should be worn for non-critical procedures such as bed making | 1 | 2 | 3 | 4 | 5 |
| 357 | Syringes and needles can be reused. | 1 | 2 | 3 | 4 | 5 |
| 358 | Any locally available sharp containers can be used. | 1 | 2 | 3 | 4 | 5 |
| 359 | Sharp collection materials:- |  |  |  |  |  |
|  | 1. Safety box/ card box/ | 1 | 2 | 3 | 4 | 5 |
|  | 2.Plastic pail with lid | 1 | 2 | 3 | 4 | 5 |
|  | 3.Plastic pail without lid | 1 | 2 | 3 | 4 | 5 |
|  | 4.Any open containers | 1 | 2 | 3 | 4 | 5 |
| 360 | Wastes can be reused. | 1 | 2 | 3 | 4 | 5 |
| 361 | Some wastes can be managed without using personal protective equipments | 1 | 2 | 3 | 4 | 5 |

| S/NO | Questions related to practices | Responses | | | | |
| --- | --- | --- | --- | --- | --- | --- |
|  |  | Never=1 | Seldom=2 | Sometimes=3 | Often=4 | Very often=5 |
| 362 | How often do you wash your hands? | 1 | 2 | 3 | 4 | 5 |
|  | 1 Before any contacts | 1 | 2 | 3 | 4 | 5 |
|  | 2.After any contacts | 1 | 2 | 3 | 4 | 5 |
|  | 3.In between patients | 1 | 2 | 3 | 4 | 5 |
| 363 | How frequent do you use the following? |  |  |  |  |  |
|  | 1.Antimicrobial soap | 1 | 2 | 3 | 4 | 5 |
|  | 2.Plain water | 1 | 2 | 3 | 4 | 5 |
|  | 3.Alcohol antisepsis and water | 1 | 2 | 3 | 4 | 5 |
| 364 | How frequent do you wear the following PPEs ? |  |  |  |  |  |
|  | 1.Gowan | 1 | 2 | 3 | 4 | 5 |
|  | 2.Gloves | 1 | 2 | 3 | 4 | 5 |
|  | 3.Apron | 1 | 2 | 3 | 4 | 5 |
|  | 4.Maske | 1 | 2 | 3 | 4 | 5 |
|  | 5.Others personal protective equipments | 1 | 2 | 3 | 4 | 5 |
| 365 | How often do you the following techniques to avoid needle stick or sharp injuries: |  |  |  |  |  |
|  | 1.Avoid recapping and other hand manipulation of needles | 1 | 2 | 3 | 4 | 5 |
|  | 2.Using safety boxes | 1 | 2 | 3 | 4 | 5 |
|  | 3.Avoid disassembling sharps | 1 | 2 | 3 | 4 | 5 |
|  | 4.Avoid over passing sharps with other person. |  |  |  |  |  |
| 366 | How often do you use the followings? |  |  |  |  |  |
|  | 1Incinerator | 1 | 2 | 3 | 4 | 5 |
|  | 2.Open damping | 1 | 2 | 3 | 4 | 5 |
|  | 3.Burial in the pit | 1 | 2 | 3 | 4 | 5 |
|  | 4.Damping | 1 | 2 | 3 | 4 | 5 |
| 367 | How frequently you process contaminated  instruments, gloves and other items using? |  |  |  |  |  |
|  | 1.Decontamination (soak in 0.5% chlorine solution  for 10 minute) | 1 | 2 | 3 | 4 | 5 |
|  | 2.Cleaning | 1 | 2 | 3 | 4 | 5 |
|  | 3.Sterlization | 1 | 2 | 3 | 4 | 5 |
| 368 | How frequent housekeeping and laundry personnel wear  gloves and other personal protective equipment? | 1 | 2 | 3 | 4 | 5 |
| 369 | How often housekeeping and laundry personnel  thoroughly wash all linen items including bed sheets,  surgical drapes, masks ,and gowns before reuse? | 1 | 2 | 3 | 4 | 5 |
| 370 | How often do you dispose contaminated wastes? | 1 | 2 | 3 | 4 | 5 |
